# Molecular Layer Deposition of Polyurea on Silica Nanoparticles and Its Application in Dielectric Nanocomposites

**DOI:** 10.1021/acs.jpcc.3c02732

**Published:** 2023-06-09

**Authors:** Amirhossein Mahtabani, Damiano La Zara, Minna Niittymäki, Rafał Anyszka, Ilkka Rytöluoto, Xiaozhen He, Eetta Saarimäki, Paolo Seri, Saeed Saedy, Kari Lahti, Mika Paajanen, J. Ruud van Ommen, Wilma Dierkes, Anke Blume

**Affiliations:** † Faculty of Engineering Technology, Department of Mechanics of Solids, Surfaces & Systems (MS3), Chair of Elastomer Technology and Engineering, University of Twente, 7500 AE Enschede, The Netherlands; ‡ Department of Chemical Engineering, Delft University of Technology, 2629 HZ Delft, The Netherlands; § High Voltage Engineering, Tampere University, P.O. Box 1001, FI-33014 Tampere, Finland; ∥ VTT Technical Research Centre of Finland Ltd., P.O. Box 1001, FI-33014 Tampere, Finland; ⊥ Department of Electrical, Electronic and Information Engineering “Guglielmo Marconi”, University of Bologna, 40136 Bologna, Italy

## Abstract

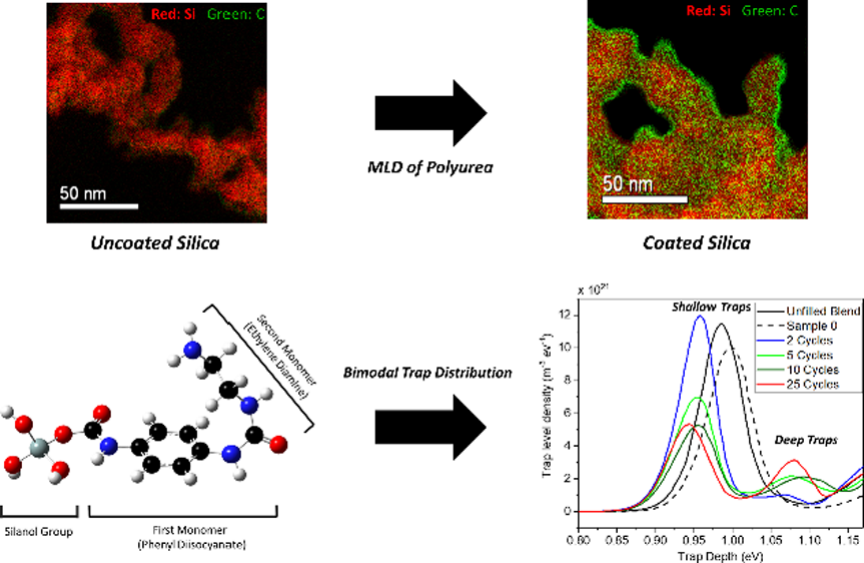

Polymer nanocomposites (NCs) offer outstanding potential for dielectric applications including insulation materials. The large interfacial area introduced by the nanoscale fillers plays a major role in improving the dielectric properties of NCs. Therefore, an effort to tailor the properties of these interfaces can lead to substantial improvement of the material’s macroscopic dielectric response. Grafting electrically active functional groups to the surface of nanoparticles (NPs) in a controlled manner can yield reproducible alterations in charge trapping and transport as well as space charge phenomena in nanodielectrics. In the present study, fumed silica NPs are surface modified with polyurea from phenyl diisocyanate (PDIC) and ethylenediamine (ED) via molecular layer deposition (MLD) in a fluidized bed. The modified NPs are then incorporated into a polymer blend based on polypropylene (PP)/ethylene-octene-copolymer (EOC), and their morphological and dielectric properties are investigated. We demonstrate the alterations in the electronic structure of silica upon depositing urea units using density functional theory (DFT) calculations. Subsequently, the effect of urea functionalization on the dielectric properties of NCs is studied using thermally stimulated depolarization current (TSDC) and broadband dielectric spectroscopy (BDS) methods. The DFT calculations reveal the contribution of both shallow and deep traps upon deposition of urea units onto the NPs. It could be concluded that the deposition of polyurea on NPs results in a bi-modal distribution of trap depths that are related to each monomer in the urea units and can lead to a reduction of space charge formation at filler-polymer interfaces. MLD offers a promising tool for tailoring the interfacial interactions in dielectric NCs.

## Introduction

Nanocomposites (NCs) for dielectric applications have shown immense potential for improving the performance in a wide range of applications, such as high-voltage electrical insulation systems, electronics, and sensors.^1−5^ The large interfacial areas introduced by the addition of a nano-material such as nanoparticles (NPs) to a polymeric matrix are known to be the reason for the superior performance of NCs over conventional microcomposites.^6^ These interfacial areas contain within them different types of physical and chemical interactions which ultimately control the macroscopic dielectric properties of the material.^7,8^ For insulating NCs, properties such as permittivity, conductivity, breakdown strength, space charge accumulation, and tracking resistance have shown to be dependent, to a great extent, on the characteristics of the interfacial areas in NCs.^2,9,10^ Therefore, attempting to control the interactions between NPs and polymer matrix is of undeniable importance in order to develop high-performance NCs for dielectric applications.

Insulating nanodielectrics have been studied extensively over the past few decades by incorporating various types of nanofillers into different polymer matrices.^11−15^ Moreover, NPs have been subjected to surface modification in order to enhance their interfacial interactions with the polymer chains and achieve better dispersion within the matrix.^16−18^ Both, the addition of NPs and their modification introduce new localized states in the material and alter the distribution of space charge.^19,20^ The presence of these localized states, i.e., traps, depending on their depth and density, can be beneficial for the dielectric properties of the NCs.^21,22^ Shallow traps, with energy levels generally well below 1 eV, can significantly hamper the formation of permanent space charge in the material as they would assist the transport of charge carriers and prevent them from accumulation.^21^ Deep traps, with energies above 1 eV, can effectively immobilize charge carriers, reduce the hopping conduction of electrons, and cause the formation of homocharge near the electrodes.^22^ For instance, it has been shown that functionalization of NPs with polar moieties can induce trapping states at the filler-polymer interfaces, reduce the charge carrier mobility and accumulation under electric fields, and ultimately enhance the dielectric properties of the corresponding NCs.^23−27^ Therefore, engineering the density and depth of traps in the material is crucial for designing high-performance insulating nanodielectrics. This can be realized by grafting functional groups with different electronic structures onto the NPs, introducing new localized states with different energy levels compared to the bare NP.

Functionalization of NPs is often performed in the liquid phase, which despite the relative effectiveness, brings about multiple issues such as solvent recovery, long operation times, high costs, severe pollution, and low efficiency.^28^ Moreover, controlling the thickness and conformity of the deposited functional layer is difficult with liquid-phase methods. In this regard, gas-phase methods offer remarkable advantages.^29^ Molecular layer deposition (MLD) is a robust technique to deposit organic films on flat surfaces as well as NPs from gaseous precursors.^30−34^ As opposed to the conventional chemical vapor deposition (CVD), MLD utilizes self-limiting reactions between bi-functional precursors to grow organic films on the solid surface in a controlled layer-by-layer fashion by dosing sequential pulses of each precursor into the reactor.^35^ The controllability of MLD enables conformal reproducible depositions on the NPs, which is of great importance in nanocomposite applications where the interface between the NP and the host polymer matrix determines the performance of the material.^36^ For instance, engineering the interfacial properties was proven to have significant influences on the charge trapping and transport mechanisms in insulating polymer NCs.^37−39^ In this regard, chemical grafting of electrically active moieties, such as amine, carbonyl, and phenyl groups on the surface of NPs, can alter the electronic structure of the filler-polymer interfaces. This can suppress space charge accumulation, enhance dielectric strength, and optimize electrical conductivity of the insulation materials.^40^ Accordingly, aromatic polyurea is a good candidate to be deposited on the NPs as its backbone contains amide and phenyl functional groups susceptible to alter the electronic structure of the filler surface in favor of the dielectric properties. While MLD of polyurea has already been performed on flat silica substrates,^41−45^ it has not yet been applied on NPs in fluidized beds.

In this study, polyurea films are deposited from phenyl diisocyanate (PDIC) and ethylenediamine (ED) onto fumed silica NPs using fluidized bed MLD. The reaction scheme and mechanism of urea formation from diisocyanate and diamine have already been demonstrated in the literature.^41,42,45−48^ After verifying the MLD growth of the polyurea film, the resulting NPs are incorporated into a polymer blend based on polypropylene (PP)/ethylene-octene-copolymer (EOC), and the dielectric properties of the NCs are investigated. First, we demonstrate the alterations in the electronic structure of silica upon depositing urea units using density functional theory (DFT) calculations. The NP dispersion quality in the polymer matrix and the crystallization behavior of the NCs are analyzed by scanning electron microscopy (SEM) and differential scanning calorimetry (DSC). Subsequently, the effect of urea functionalization on the charge trapping and transport under a direct current (DC) electric field is studied using thermally stimulated depolarization current (TSDC) method. In addition, in order to further analyze the relaxation processes and the dielectric response of the NCs, broadband dielectric spectroscopy (BDS) is performed and the results are discussed.

## Experimental Section

### Materials

AEROSIL 200 silica NPs (primary particle size of 12 nm, specific surface area of ∼200 m^2^/g), with high purity and low moisture content, were received from Evonik Industries (Germany). The NPs were kept in an oven over night at 120 °C to remove the physisorbed water. PDIC (97%) and ED (99%) (Sigma-Aldrich, Germany) were used without any additional purification. Precursors were weighed and placed into stainless-steel bubblers inside a glove box under inert environment. Mineral oil Kaydol (Sonneborn, USA) and sodium hydroxide (ABCR, Germany) were used in washing bubblers in the MLD setup. KBr (99 + %, FTIR grade, Harrick Scientific Corporation) was used as received to prepare samples for DRIFTS measurements.

### Methods

#### MLD Experiments

The MLD experiments were performed in a fluidized bed reactor operating at atmospheric pressure. The reactor included a glass column with an internal diameter of 2.5 cm and height of 25 cm placed on top of a Paja PTL 40/40-24 vibration table to enhance the fluidization of the silica NPs. In order to homogenize the gas flow through the column and to avoid any powder loss from the top of the reactor, two stainless-steel distributor plates were placed at the two ends of the column. The precursors were kept in separate stainless-steel bubblers, under inert nitrogen atmosphere, and heated using a heating tape. PDIC is a low-vapor-pressure precursor (<0.0075 Torr at 20 °C and 14.25 Torr at 118 °C) and solid below 96 °C. So, the bubbler was heated to 110 °C to increase the vapor pressure. The ED bubbler was kept at room temperature due to its high vapor pressure (10 Torr at 20 °C). Precursors were carried into the reactor by a nitrogen flow (99.999 v/v%) passing through the bubblers. Three separate gas lines, two for precursor dosing and one for the nitrogen purge, were connected to the reactor. This arrangement ensures pure dosing of each precursor and eliminates unwanted reactions inside the lines. The lines were heated to and kept at 140 °C to prevent condensation and under-delivery of gaseous precursors. The reaction temperature in the column was set at 150 °C by means of a feedback-controlled infrared lamp, mounted parallel to the column. The bed temperature was monitored using a type-K thermocouple inserted into the bottom of the column. The exiting gas was sent from the top of the reactor through a series of washing bubblers, containing mineral oil (Kaydol) and sodium hydroxide (NaOH), to capture unreacted precursors and process byproducts. All the reactor constituents were contained inside a closed cabinet. In each experiment, 2 g of SiO_2_ powder was placed in the column. Each MLD cycle started with a PDIC pulse, followed by an ED pulse through the fluidized bed, with nitrogen purging in between and at the end. The pulse times were optimized to reach surface saturation at a constant number of five cycles. This was done by varying the pulse time of one precursor while keeping the other at a constant value. After verifying the MLD saturation behavior, the growth of the deposited film was analyzed after 2, 5, 10, and 25 cycles with the optimal pulse times of 1 min for each precursor with 5 min N_2_ purging in between.

#### Thermogravimetric Analysis (TGA)

**Figure 1:**
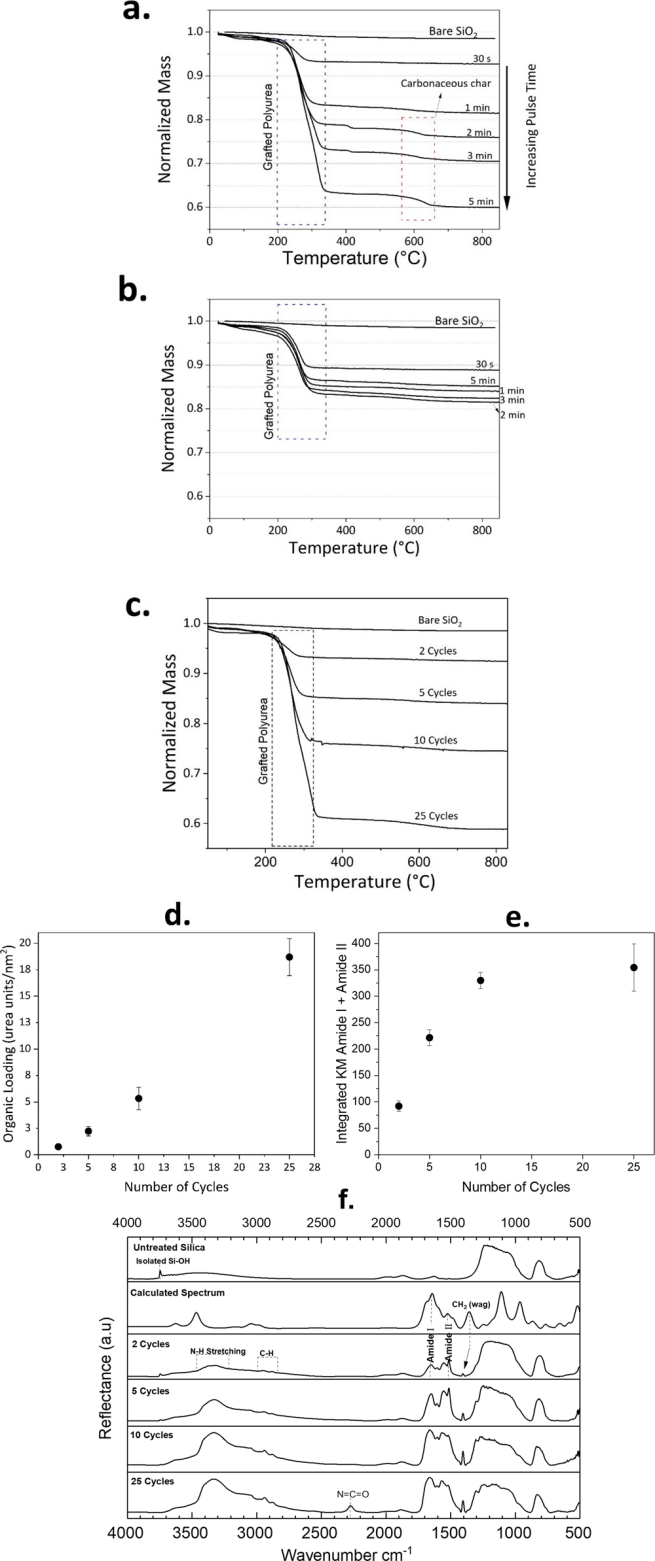
Thermograms of modified silica NPs with different (a) PDIC (ED constant at 2 min) and (b) ED (PDIC constant at 1 min) pulse times, and (c) different number of cycles (with PDIC and ED saturation pulse times of 1 min), alongside with the thermogram of the bare SiO_2_ NPs. (d) Polyurea loading with respect to the number of cycles calculated from TGA. (e) Integrated Kubelka–Munk (KM) amide I and amide II bands with respect to the number of cycles. (f) Spectra of the polyurea coated NPs with different MLD cycles compared to the untreated silica and the DFT calculated spectrum.

In order to verify the MLD growth and quantify the polyurea coating, TGA was performed using a TGA/SF1100 STARe system (Mettler Toledo, USA). The measurements were done by heating 2–3 mg of the fine powder from room temperature to 850 °C with a rate of 20 °C/min under nitrogen atmosphere while monitoring the mass of the sample. We considered the mass loss in the temperature range of 200–350 °C to be related to the removal of polyurea chains (see Figure 1). With this assumption, one can calculate the polyurea loading (υ), from the TGA mass loss, using eq 1.
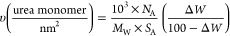
1where υ is the polyurea loading as the number of urea segments deposited per unit surface area, Δ*W* is the TGA mass loss between 200 and 350 °C, *M*_W_ is the molecular weight of a single urea segment from one PDIC/ED cycle (220 g/mol), *S*_A_ is the average surface area of the NPs (200 m^2^/g), and *N*_A_ is the Avogadro constant.

#### Diffuse Reflectance IR Fourier Transform Spectroscopy (DRIFTS)

To confirm the polyurea deposition and its self-limiting growth, diffuse reflectance IR Fourier transform spectroscopy (DRIFTS) was utilized. Analyses were conducted using a Perkin Elmer Spectrum 100 spectrometer (USA) equipped with a DRIFTS accessory. The samples were prepared using KBr as background. Spectra were recorded from 4000 to 400 cm^–1^ and averaged over 128 scans, using a resolution of 4.0 cm^–1^. All the tests were performed at room temperature.

#### X-ray Photoelectron Spectroscopy (XPS)

XPS was conducted by means of a PHI Quantera scanning X-ray microscope (USA). By irradiating a material with a beam of X-rays, while simultaneously measuring the kinetic energy and number of electrons that escape from the surface (up to 10 nm in depth), XPS can provide accurate information about the chemical composition of the deposited film. Using this method, it is also possible to evaluate the type of bonds each element is involved in.

#### Preparation of the NCs

NCs based on polypropylene/ethylene-octene copolymer (PP/EOC) blends filled with functionalized silica NPs were prepared by mixing 1 wt % of the silica as well as an antioxidant package into the polymer matrix in a twin-screw micro extruder (Haake MiniLab Rheomix CTW5, Thermo Fisher Scientific, Waltham, Massachusetts, USA) at 230 °C and 100 rpm, and subsequent injection of the molten compound into a square 26 × 26 × 0.5 mm mold at 60 °C using a Haake MiniJet Pro Piston Injection Molding System (Thermo Fisher Scientific, Waltham, Massachusetts, USA).

#### Morphology and Crystallinity Analysis

SEM was performed on the dielectric NCs using a Jeol JSM-6400 (Jeol Ltd., Tokyo, Japan) to analyze the filler dispersion and polymer blend morphology. In order to achieve a higher resolution and visualize the particles more clearly, SEM was performed on the samples, with and without gold sputtering. The silica agglomerate size distribution in the NCs was analyzed using the open-source ImageJ software with Trainable Weka Segmentation plugin.^49^ DSC was performed by means of a DSC 2500 (TA Instruments, USA). Specimens were subjected to two heating/cooling cycles from −70 to 200 °C in nitrogen atmosphere. The heating/cooling rate was set at 3 °C/min in order to match the heating rate in TSDC measurements (explained in the next section).

#### Thermally Stimulated Depolarization Current (TSDC)

TSDC was utilized to analyze the charge trapping and transport phenomena in the NCs under a high DC field. 100 nm thick circular gold (Au) electrodes were deposited on both sides of each NC specimen by e-beam evaporation under high vacuum. Subsequently, they were short-circuited to remove residual charges and kept in a vacuum desiccator overnight prior to the measurements. The TSDC tests were carried out by heating up the NC specimens rapidly to the poling temperature of 70 °C, and then a 3 kV/mm DC electric field was applied for 20 min under isothermal conditions. The samples were then rapidly cooled down to −50 °C while the electric field was still on. This would force the polarized species and injected charges to remain in the specimen. Next, the samples were short-circuited and linearly heated up to 140 °C at 3 °C/min, while the depolarization current was being measured by an electrometer (6517B Keithley Instruments, Cleveland, Ohio, USA).

#### Broadband Dielectric Spectroscopy (BDS)

Dielectric spectroscopy was performed on the NCs after the TSDC tests, and the real (ε_r_′) and imaginary (ε_r_″) parts of complex relative permittivity (as in eq 2) were measured using a Novocontrol Alpha-A dielectric analyzer (Montabaur, Germany) in a broad frequency range of 10^–2^ to 10^6^ Hz and under an applied voltage of 1 V. All the measurements were done at ambient temperature.
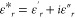
2

#### Computational Details

DFT calculations were carried out on two model structures: a silica protoparticle with silanol groups on all sides and an urea segment from the reaction between PDIC and ED grafted to a silanol group. The models were prepared using GaussView 6, and the quantum mechanical calculations were done by the Gaussian 09 W package.^50^ The ground state geometry of each model at their minimum potential energy was calculated by the solution of the time-independent Schrödinger equation, utilizing the hybrid Becke three-parameter exchange correlation functional (B3LYP), with a split-valence double zeta basis set 6-31 + G(d) to include both polarization functions and long-range interactions.^51−53^ All calculations were performed considering room temperature and in the presence of an external electric field of 0.0001 au (∼51.4 KV/mm). Total density of states (TDOS) and the band structure of the models were calculated by means of the multifunctional wavefunction analyzer Multiwfn.^54^

## Results and Discussions

The key characteristic of an MLD process is the self-limiting, layer-by-layer growth of the organic film. We evaluated the possibility of depositing polyurea, in a self-saturating fashion, on silica NPs. TGA and DRIFTS were utilized to verify this self-limiting behavior and to quantify the amount of deposited organic matter on the NPs. The TGA thermograms of the NPs modified with different precursor pulse times, along with that of the untreated silica, are presented in Figure 1a,b.

The major mass loss between 200 and 350 °C can be attributed to the removal of polyurea chains from the NP surface. For PDIC pulse times longer than 1 min, there is an additional mass loss step between 550 and 650 °C related to the decomposition of carbonaceous char produced during the first decomposition step.^55,56^ This is more likely when there is an abundance of aromatic groups in the film due to the physisorption of PDIC, which is also supported by the emergence of the isocyanate band at 2280 cm^–1^ in the DRIFTS spectrum in case of 5 min PDIC pulse time (Figure S1). This suggests that after 1 min of PDIC pulsing, the growth is dictated by the physisorption of unreacted precursor that results in additional mass loss in TGA thermograms. This is a common case for low-vapor-pressure precursors such as PDIC for which a significantly longer purging time or higher reaction temperatures would be required to remove the unreacted molecules. Nevertheless, the DRIFTS spectra show the saturation of the amide I and amide II bands, characteristic of the urea segments, emerging at 1650 and 1510 cm^–1^, respectively (see Figure S1), after 1 min of PDIC exposure. This indicates a self-limiting reaction, typical of the MLD process, even though characterized by physisorption of unreacted PDIC for exposure times above the saturation point.^31,42,57^ Utilizing the saturation pulse times of each precursor, the evolution of the polyurea film was studied with respect to the number of cycles (Figure 1c). Figure 1d,e verifies the linear growth of the organic loading on the NPs at least up to 10 MLD cycles. Figure 1f demonstrates the DRIFTS spectra for the polyurea coated NPs in different cycles compared to the untreated silica and the DFT calculated spectrum. All the bands related to the polyurea film, i.e., the amide I and amide II modes, the N–H and C–H stretch, and the CH_2_ wagging, increase significantly upon increasing the number of MLD cycles. At 25 cycles, a band emerges at 2280 cm^–1^ related to the isocyanate groups and is indicative of unreacted PDIC molecules that were not purged from the reactor. This would naturally disturb the MLD linear growth at 25 cycles as it is observed comparing Figure 1d,e. It is also noteworthy that the isolated silanol band at 3750 cm^–1^ completely vanishes after 10 MLD cycles, which is due to their chemical attachment to the urea groups. Comparing the experimental and the DFT calculated spectra, except for a few discrepancies due to the DFT model simplification, bands are in good accordance with the experimental spectra. These differences between the model and experimental spectra stem from the intramolecular interactions in the film. For instance, the N–H stretching band observed at 3400 cm^–1^ in the calculated spectrum has been red-shifted and broadened in the experimental spectra. This is likely due to the different levels of hydrogen bonding between the N–H groups and the carbonyl oxygen of the adjacent chains, which has also been reported in previous studies on polyurea films.^42,58^

**Figure 2:**
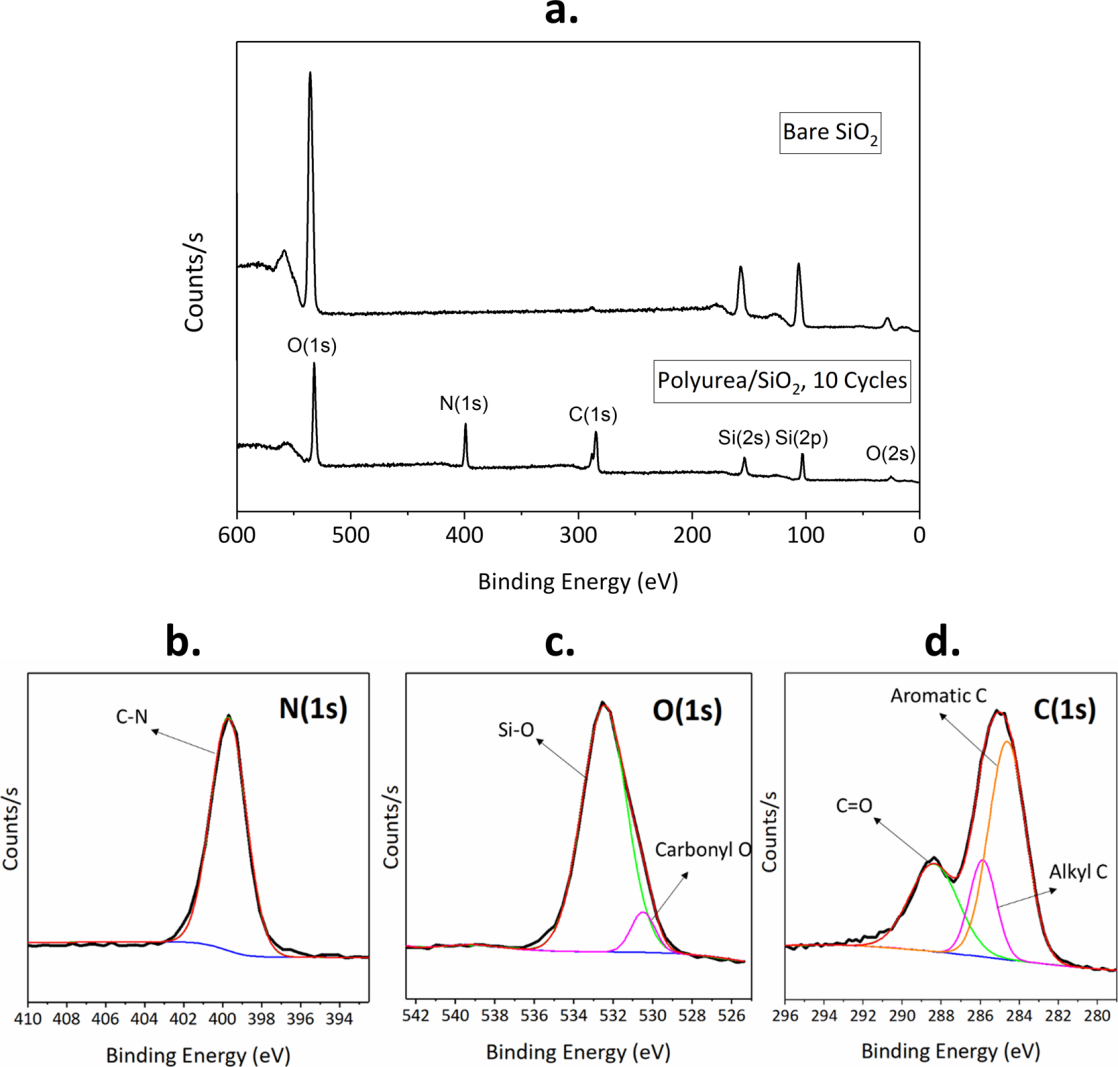
(a) XPS full spectra of the NPs treated in 10 cycles compared to the untreated reference and (b–d) deconvoluted N(1s) and O(1s) and C(1s) elemental fine scans for the silica sample treated in 10 cycles.

The XPS spectra of the modified silica after 10 MLD cycles is compared to the bare SiO_2_ in Figure 2. The emergence of N(1s) and C(1s) bands for the treated NPs in Figure 2a confirms the presence of the polyurea chains on their surface. The atomic percentages presented in Table 1 show the gradual increase of carbon and nitrogen contents with the number of cycles. For an ideal polyurea film from PDIC/ED, the stoichiometric C:N:O ratio should be 5:2:1. For the 10 cycle sample, the C:N:O ratio reaches 4.7:1.8:1, which signifies the close to ideal coverage of polyurea film on the NPs after 10 cycles. The C:N ratio is in good accordance with the theoretical ratio of 2.5 for an urea segment, regardless of the number of cycles. The elemental fine scans (Figure 2b–d) verify the chemical composition of the polyurea film. The N(1s) scan exhibits one single peak at 399.7 eV, representative of the C–N bonds in the urea segments. The O(1s) fine scan is deconvoluted into two peaks at 530.4 and 532.5 eV, which are indicative of oxygen atoms in the carbonyl groups and silica, respectively. The C(1s) fine scan reveals three peaks: the one with the lowest binding energy at 284.4 eV can be attributed to the carbons from the electron-rich aromatic groups. The peak at 285.7 eV results from the alkyl carbons, and the highest binding energy peak at 288.5 eV is indicative of carbons from the carbonyl groups. The theoretical ratio of the three types of carbon, i.e., aromatic/alkyl/carbonyl in the urea segments, is 3:1:1, and our deposited film at 10 cycles exhibits a ratio of 2.1:1.1:1, which is in range with the theoretical ratio. The shortage of the aromatic carbons compared to the theoretical ratio is likely due to the growth of the urea groups from the aromatic rings. The substituted carbons are, therefore, chemically shifted by the urea toward higher binding energies, which leads to the expected peak ratio to be 2:2:1 rather than 3:1:1.^42^ It is noteworthy that the fine scans were performed only on the 10-cycle sample to ensure that no relevant amount of unreacted PDIC is present on the surface.

**Table 1:** Atomic Percentages of C, N, O, C:N:O, and C:N Ratios for NPs Treated at Different Number of Cycles

Number of MLD cycles	C (atom %)	N (atom %)	O (atom %)	Si (atom %)	C:N:O^a^	C:N
2	7.01 ± 0.08	2.81 ± 0.23	62.69 ± 0.47	27.38 ± 0.42	2.5:1:2.8	2.49
5	17.96 ± 0.57	7.33± 0.21	52.06 ± 0.43	22.61 ± 0.03	2.6:1.07:1	2.45
10	27.87 ± 0.12	11.71 ± 0.82	42.25 ± 0.35	18.16 ± 0.82	4.7:1.8:1	2.38
25	44.42 ± 5.16	17.79 ± 2.17	27.37 ± 4.55	10.41 ± 2.8	6.8:2.7:1	2.5

a The C:N:O ratio is calculated after subtracting the oxygen content of SiO_2_.

**Figure 3:**
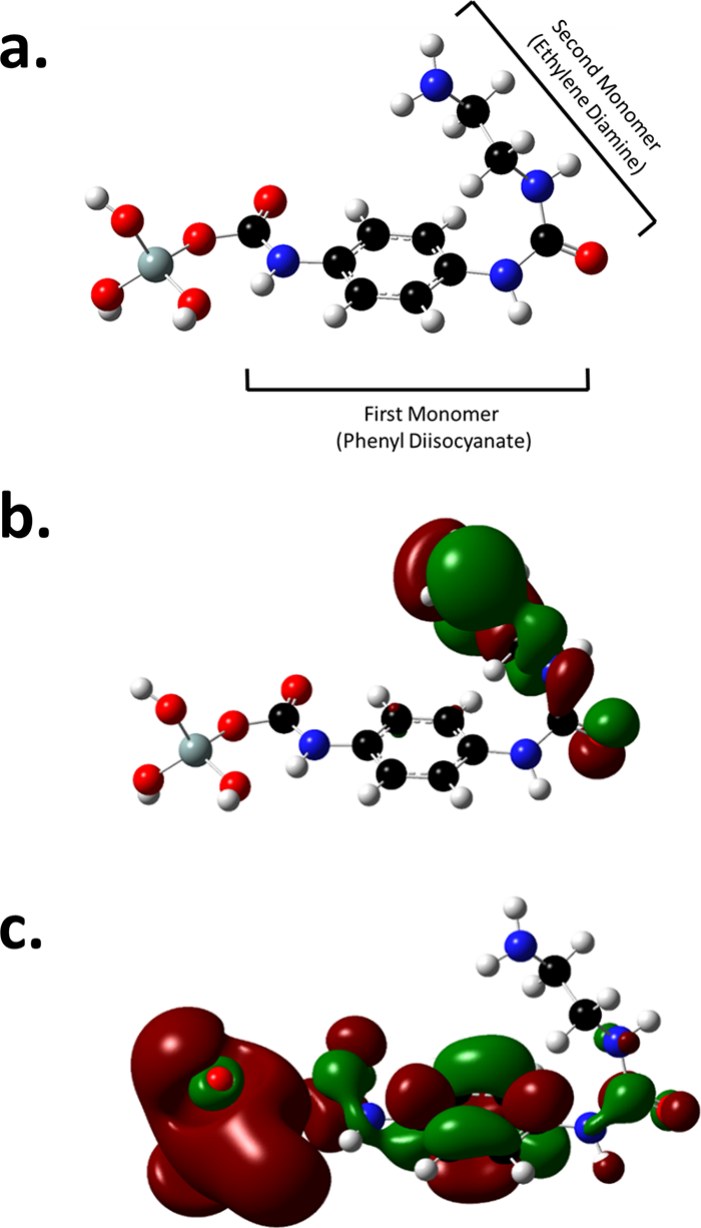
(a) Optimized geometry of an urea unit made of phenyl diisocyanate and ethylene diamine grafted to a silanol group on silica; (b) highest occupied molecular orbitals (HOMO) and (c) lowest unoccupied molecular orbitals (LUMO) visualized for the modified silica (gray: silicon, red: oxygen, black: carbon, blue: nitrogen, and white: hydrogen; the red and green colors of the MOs are related to the positive and negative wavefunctions, respectively).

As it was pointed out earlier, grafting electrically active functional groups onto the surface of NPs would potentially alter the electronic structure of the surface groups and create localized states at the filler-polymer interfaces. To demonstrate these alterations upon grafting urea films to the silica surface, DFT calculations were performed on a model structure of an urea unit coupled to a silanol group (see Figure 3a). Figure 3b,c demonstrates the distribution of the highest occupied molecular orbitals (HOMO: −7.05 eV) and lowest unoccupied molecular orbitals (LUMO: 0.86 eV) of this structure, respectively. It is evident that the HOMO are mostly located on the second monomer ED, whereas the LUMO are distributed over the first monomer phenyl diisocyanate (PDIC) as well as the dangling silanol groups in the model. This suggests, on the one hand, that the silanol groups on the silica surface can noticeably contribute to the density of unoccupied states, i.e., it is rather likely to find conducting electrons around them. Therefore, grafting other moieties to cover these surface groups in order to control conductivity and space charge phenomena in the final NC appears very relevant. On the other hand, between the two monomers in the urea repeating unit, PDIC is more susceptible to contribute to the conduction processes in the presence of an electric field, likely by creating shallow trapping states. Moreover, the presence of the HOMO around the ED monomer can be beneficial in hampering the formation of space charge in the presence of the DC electric field. This is similar to what we observed for amino-modified silica, where the amino group’s large density of occupied states hindered the formation of space charge at the silica-polymer interfaces.^23^

**Figure 4:**
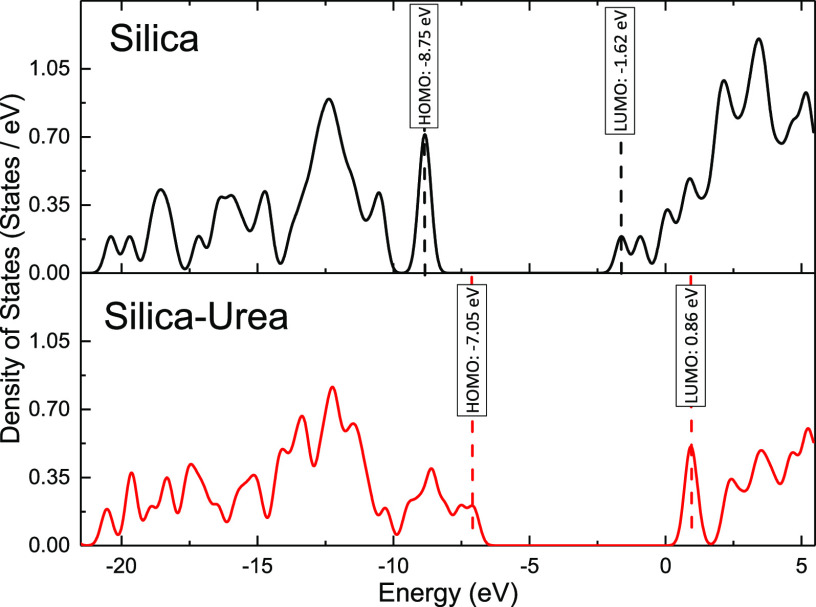
Total density of states (TDOS) for the urea-silica structure compared to the neat silica.

The TDOS at different energy levels for the bare as well as the urea modified silica are depicted in Figure 4. For the unmodified silica, a rather large density of hole traps is observed at the tail of the valence band, inside the band gap. Furthermore, at the bottom of the conduction band, there is a distribution of relatively shallow electron traps. By introducing the phenyl and amide groups to the silica, both valence and conduction band extrema shift to higher energy levels and the band gap becomes slightly (0.84 eV) broader. The density of electron traps is larger for the urea modified silica, implying that new localized states are created as a result of this modification. These states are extended over 1.4 eV below the conduction band, suggesting that there are contributions of both shallow and deep traps from this urea unit. The density of conduction states is significantly lower for the urea modified silica, which suggests that the conducting electrons are less likely to be drifting in the conduction band, when this urea unit is present on the silica surface.

**Figure 5:**
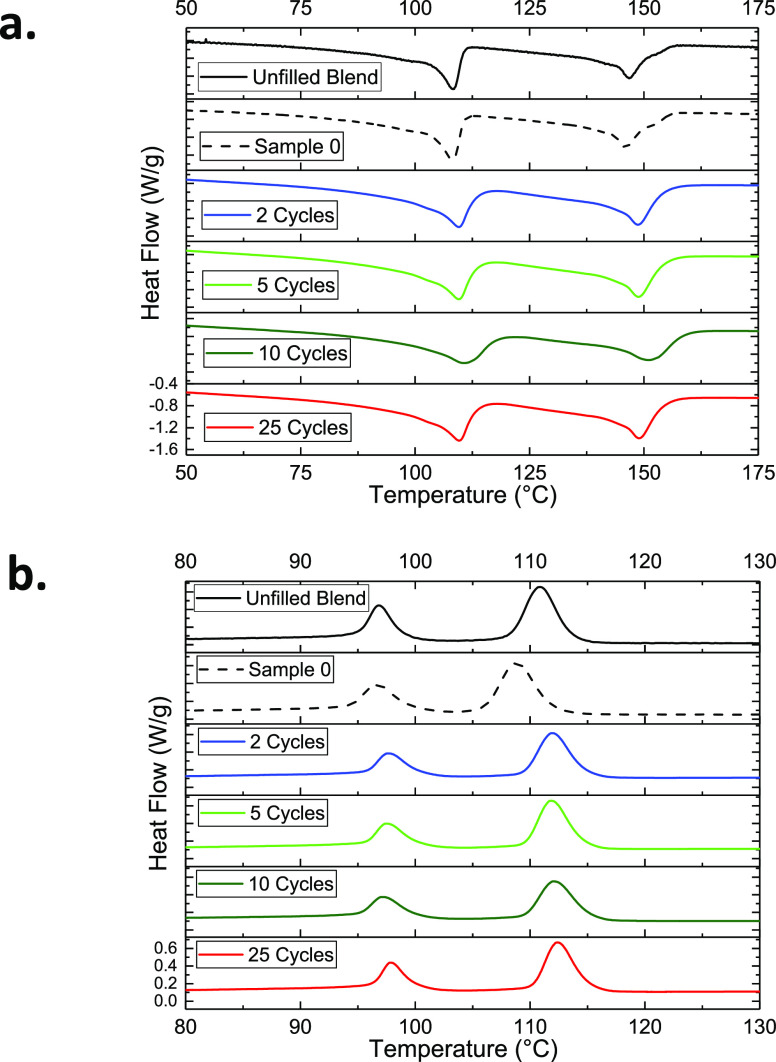
(a) Melting and (b) crystallization behavior of the studied NCs compared to the unfilled blend.

Surface characteristics of the NPs can directly affect their nucleating ability in the polymer matrix and hence the crystalline structure of the NCs. Consequently, this can influence the dielectric response of the material. Therefore, DSC was performed to analyze and compare the crystallization behavior of the NCs. Figure 5a,b represents the melting and crystallization curves for the unfilled blend and NCs with and without filler functionalization. The DSC spectra of the blend consist of four distinct peaks: two endothermic peaks (108 and 148 °C) and two exothermic peaks (97 and 115 °C) corresponding to the melting and crystallization temperatures of the EOC and PP domains, respectively. The melting curves (Figure 5a), on the one hand, exhibit no significant changes upon incorporation of the silica and functionalization of it. On the other hand, the onset of crystallization tends to decrease by 3 °C when the bare NPs are added to the system. This is despite the expected nucleating effect of NPs in composite systems and is likely due to the adsorption of polar antioxidants onto the silica surface, reducing heterogeneous nucleation during the cooling step.^23,38,59^ Nevertheless, the modified NCs exhibit crystallization onsets similar to that of the unfilled blend. This implies that the polyurea deposition facilitates nucleation. Moreover, all the MLD modified samples exhibit similar crystallization curves indicating that the amount of deposited polyurea has no significant effect on the nucleation and crystal growth in the NCs. The degree of crystallization for each sample is calculated from the enthalpy of melting and presented in Table 2. It is evident that the addition of NPs and their modification does not influence the overall degree of crystallization in the NCs. Accordingly, it can be assumed that any discrepancy in the dielectric response of the studied NCs would not be influenced by the differences in crystallization behavior of the materials.

**Table 2:** DSC Parameters of All Studied Samples

	Onset of crystallization (°C)	Enthalpy of melting (J/g)	Degree of crystallinity (%)
Unfilled Blend	115	77.3	31
Sample 0	112	74.9	30
2 cycles	114	72.1	29
5 cycles	115	75.1	30
10 cycles	115	71.6	29
25 cycles	115	73.2	29

**Figure 6:**
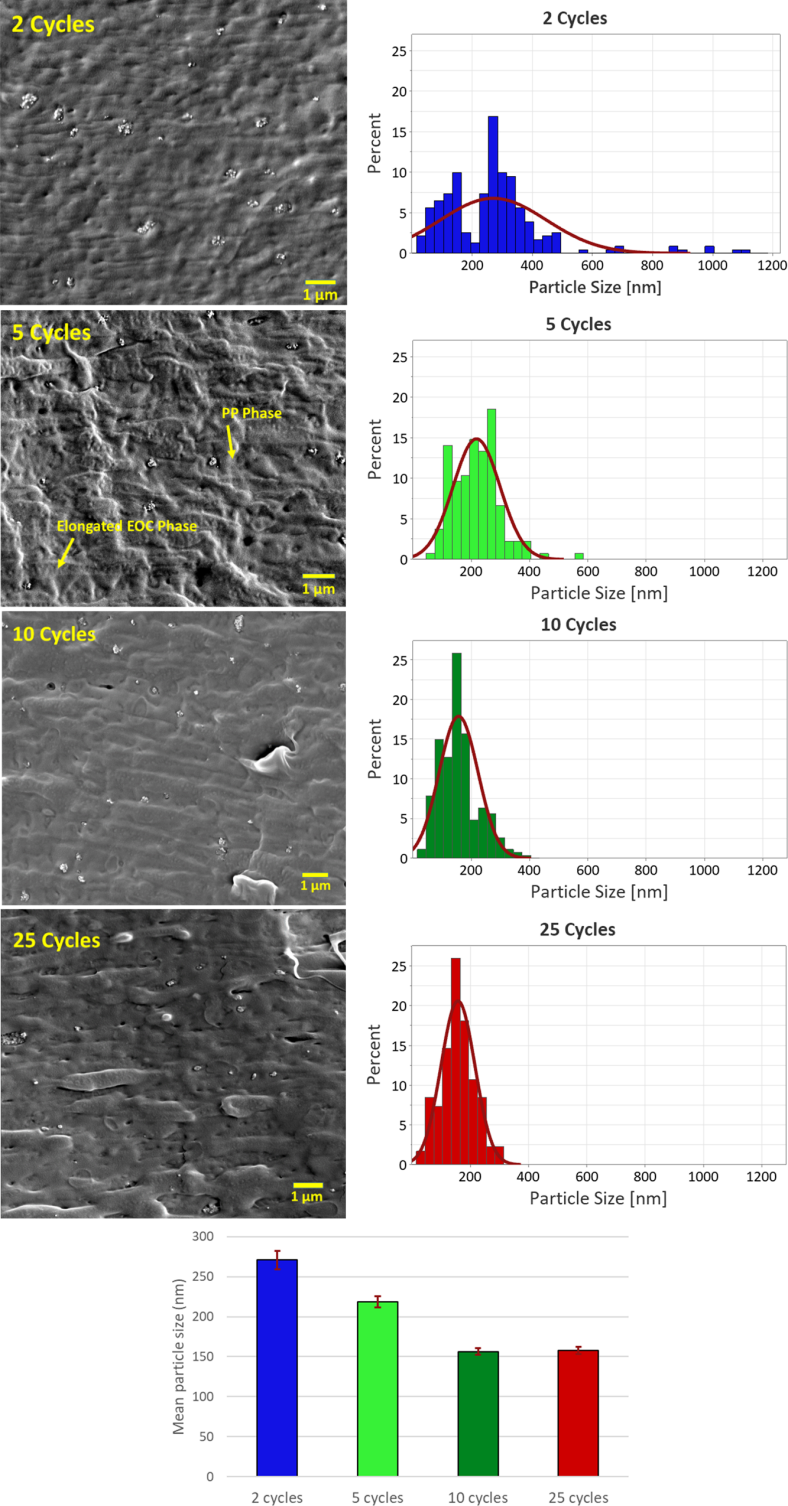
SEM images of NCs containing MLD modified silica, along with the particle size histograms and a plot of mean particle sizes. The image of the five cycle sample is taken without gold sputtering in order to visualize the phase distribution of the polymer blend.

In order to analyze the dispersion of NPs in the polymer matrix, SEM was performed on the NCs and the results are presented in Figure 6. In order to achieve a higher resolution and visualize the particles more clearly, SEM was performed on these samples with and without gold sputtering. In case of the sputtered samples, however, the morphological details of the polymer matrix were masked. Therefore, we present the image of the five cycle sample without sputtering in order to discuss the morphology of the polymer blend. It can be observed that the PP/EOC blend exhibits a two-phase morphology with the EOC domains elongated in the direction of the flow in the mold. This was also observed in our previous study.^23^ The mean aggregate size of the untreated silica NPs was reported to be around 300 nm in PP/EOC blends.^23^ Upon increasing the number of MLD cycles, the mean aggregate size in the NCs reduces, reaching ∼160 nm for samples with 10 and 25 MLD cycles. This indicates that the surface modification has effectively enhanced the dispersion of NPs throughout the polymer matrix.

**Figure 7:**
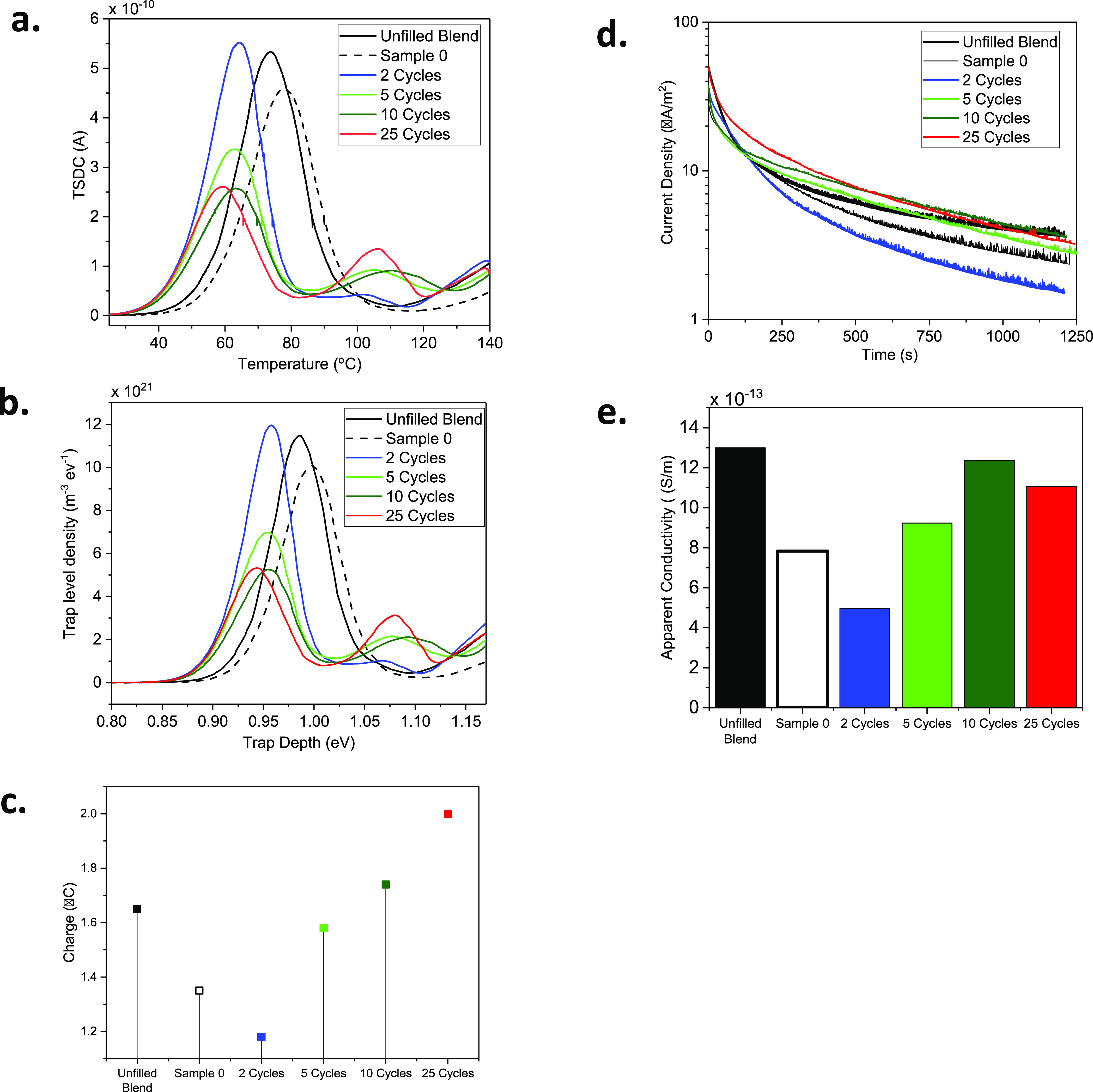
(a) TSDC spectra, (b) trap level distribution and density, (c) amount of injected charge during poling, (d) current density during poling, and (e) apparent conductivity at the end of poling for all NCs and the unfilled reference.

TSDC is a versatile method used to quantitatively analyze the charge trapping and transport phenomena in nanodielectrics by monitoring the discharge current when a charged sample is thermally stimulated. This would result in a spectrum that is able to reveal all low-frequency motions in the material.^60^ Therefore, peaks appearing above the glass transition of nonpolar material can, in general, be attributed to the space charge relaxations. Accordingly, the TSDC peak temperature and the current magnitude can be correlated with the depth and density of charge traps in the material, respectively. The TSDC spectra of all the studied systems are presented in Figure 7a. The trap depth and density distributions are numerically calculated from the TSDC data, using a method reported by Tian et al.,^61^ and presented in Figure 7b. The TSDC spectrum of the unfilled polymer blend exhibits a relaxation peak at 74 °C, indicating the presence of traps with a distribution of depths around 0.98 eV (see Figure 7b). These are likely charges released from the interfacial regions between the two polymer domains, and the crystalline/amorphous interfaces within either polymer phase. Upon incorporation of the untreated silica (sample 0), the peak trap density appears at a slightly deeper level compared to the unfilled blend. The introduction of polyurea films to the NPs has a noticeable influence on the TSDC spectra. At two MLD cycles, a rather large relaxation peak appears at 63 °C, which can correspond to the existence of relatively shallow trapping states with depths around 0.95 eV.^61^ Moreover, a smaller peak is observed at ∼105 °C indicative of deeper states with energies around 1.1 eV (below the conduction band). As the number of MLD cycles increases, the density of the deep traps increases, while the contribution of the shallow traps reduces. The DFT calculations predicted that there are contributions of traps with varying depths in the urea units under consideration. Also, the distribution of the molecular orbitals suggested that the phenyl rings and the amide groups are likely responsible for the relatively shallow and deep traps, respectively. Therefore, the lower energy peak in the TSDC spectra of the modified NCs can be attributed to the relaxation of space charge trapped in the phenyl group’s localized states, whereas the high energy peak is due to the relaxation of charges that occupy the amide deep traps. As the MLD film grows with increasing number of cycles and the density of deep traps increases, the charges would be immobilized for longer periods of time, thus hampering the trapping/detrapping processes inside the material. This would naturally result in a lower contribution of the shallower traps, and consequently more space charge may be formed at the interfaces. This can clearly be seen in Figure 7c, where the amount of injected charge is minimum at two MLD cycles with the highest density of shallow traps. It is clear that the polarization step was too short to reach steady-state DC conduction in the samples; nevertheless, Figure 7e demonstrates that the NC with two-cycle-modified silica exhibits the lowest apparent conductivity (4.9 × 10^–13^ S/m) at the end of the poling step among all studied samples.

**Figure 8:**
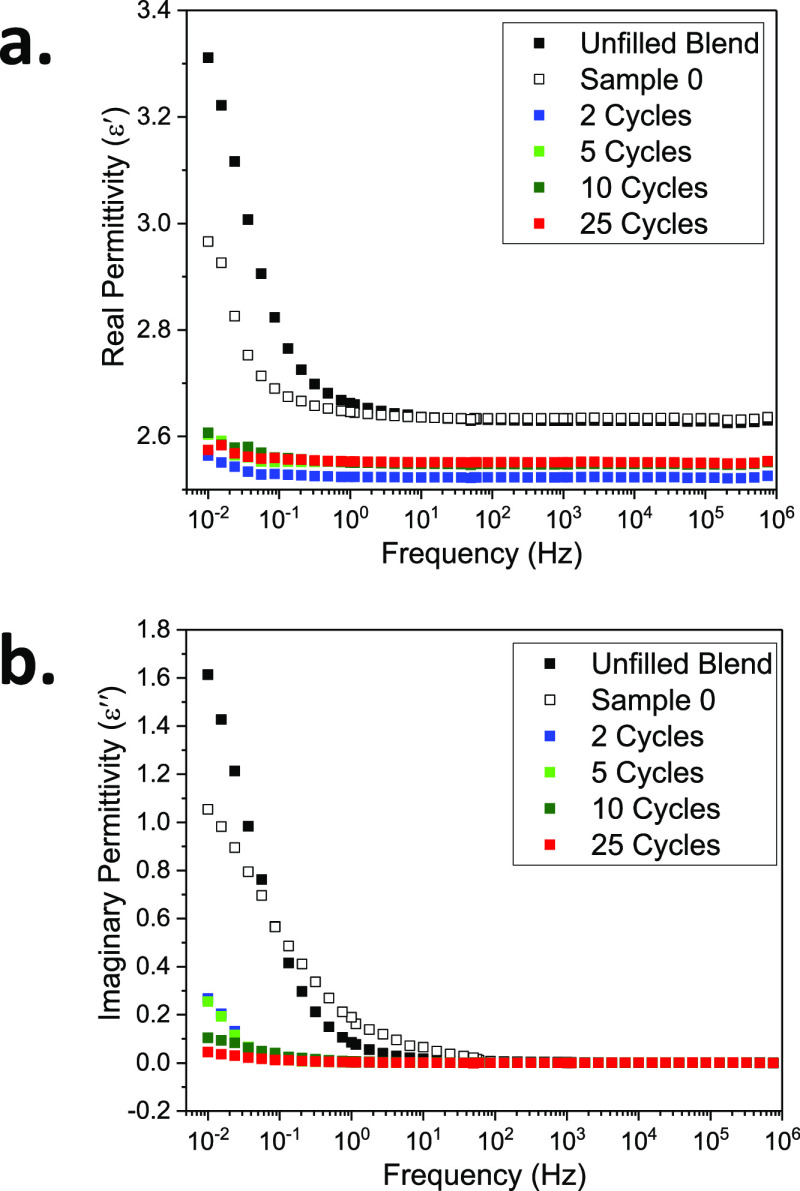
(a) Real and (b) imaginary parts of permittivity for the unfilled blend compared to NCs with modified and nonmodified silica.

BDS was performed in order to investigate the relaxation processes in the NC systems in the presence of an alternating electric field. These relaxation processes, depending on the frequency range, can then be attributed to dielectric phenomena such as polarization and space charge formation. Figure 8 presents the variations of the real (ε_r_′) and imaginary (ε_r_″) permittivity with respect to the frequency of the applied field. At frequencies higher than 1 Hz, the unfilled blend and sample 0 show little difference in their values of real permittivity. It is also clear that the modified NCs exhibit lower values of ε_r_′ at all frequencies, compared to the two references. Since real permittivity is related to the number of polarizable species in the system, the NC with two-cycle-modified silica, with the lowest grafting density of polyurea, exhibits the lowest real permittivity among all MLD modified samples. ε_r_″ remains constant for all the samples at higher frequencies. At low frequencies (generally less than 1 Hz), however, there is a steep increase in both parts of permittivity, indicative of Maxwell–Wagner space charge relaxations.^6,62^ On the one hand, it is clear that these relaxations are somewhat suppressed when the NPs are incorporated into the system. On the other hand, deposition of polyurea on the NPs has resulted in further suppression of this low-frequency rise. This indicates that the presence of polyurea films on the NPs has significant effects on the polarization processes and space charge formation in the corresponding NCs. This is well in-line with the DFT predictions: the reduced space charge relaxations in the modified NCs can be attributed to either the presence of relatively shallow traps introduced by the phenyl rings in the polyurea film or the amide groups’ occupied deep traps hampering the formation of space charge at the filler-polymer interfaces.

Evidently, by grafting a homogenous polyurea film onto the silica NPs, it is possible to create a bi-modal distribution of trap depths. The phenyl rings in the deposited film are able to introduce relatively shallow trapping states that could reduce charge mobility, and hence hinder conduction processes. Instead, the amide groups are prone to create deeper states, which in an occupied state can hamper further formation of space charge at the filler-polymer interfaces. Naturally, the lower the density of the occupied deep traps, the lower the formation of space charge. Moreover, as shown in the DFT results, the second monomer in the urea unit (ED) induces hole trap states that can further reduce the overall charge mobility in the NCs. The presence of all these newly introduced states at the filler-polymer interface makes this type of modification very suitable for developing dielectric NCs with superior insulation properties.

## Conclusions

We have successfully demonstrated the deposition of polyurea films on the surface of fumed silica NPs via MLD in a fluidized bed using PDIC and ED as the precursors. The key characteristics of an MLD process, i.e., self-limiting behavior and linear film growth rate, were verified, and the chemistry of the deposited film was studied. Subsequently, the electronic structure of silica upon deposition of polyurea films was investigated using DFT. DFT revealed the presence of both shallow and deep localized states resulting from the urea units. Also, the LUMO and the HOMO levels were distributed around the PDIC and ED monomers, respectively. This indicated that each monomer can play a different role in charge transport processes in the dielectric. Dielectric measurements revealed that the polyurea deposition results in a bi-modal distribution of deep and relatively shallow traps with densities dependent on the number of MLD cycles. In particular, with increasing number of cycles, the density of the deep traps increases, whereas that of shallow traps decreases. The large density of valence states related to the amino functional groups in the polyurea film hampered the formation of space charge, and the relatively shallow trapping states corresponding to the phenyl rings resulted in a lower current density under a DC electric field. Therefore, MLD offers a reliable tool for controlling the density and depth of charge traps in nanodielectrics, which can be of utmost importance in developing insulating materials with low space charge accumulation and conductivity.

## Supporting Information Available

Saturating behavior of the polyurea film growth after five MLD cycles; saturation curves from TGA: polyurea film growth at different precursor pulse times; DRIFTS spectra for the saturation experiments with different precursor pulse times; saturation curves from DRIFTS: integrated Kubelka–Munk (KM) amide I and amide II bands; and carbon and silicon mapping from TEM images which demonstrate the spatial distribution of the deposited film on silica aggregates (PDF)
