# Supplementary material for: Molecular Layer Deposition of Polyurea on Silica Nanoparticles and Its Application in Dielectric Nanocomposites
Source: J Phys Chem C Nanomater Interfaces. 2023 Jun 9;127(24):11736–47. doi: 10.1021/acs.jpcc.3c02732 (PMC10291639; doi:10.1021/acs.jpcc.3c02732)
Supplement: Supplementary file 1 — jp3c02732_si_001.pdf [file jp3c02732_si_001.pdf]

Supporting Information

Molecular Layer Deposition of Polyurea on Silica Nanoparticles and Its Application in Dielectric Nanocomposites

Amirhossein Mahtabani[1], Damiano La Zara[2], Minna Niittymäki[3],

Rafal Anyszka[1], Ilkka Rytöluoto[4], Xiaozhen He[1], Eetta Saarimäki[4], Paolo Seri[5], Saeed Saedy[2], Kari Lahti[3],

Mika Paajanen[4], J. Ruud van Ommen[2], Wilma Dierkes[1*], Anke Blume[1]

[1]University of Twente, Faculty of Engineering Technology, Department of Mechanics of Solids, Surfaces & Systems (MS3), Chair of Elastomer Technology and Engineering, 7500 AE Enschede, The Netherlands

[2]Department of Chemical Engineering, Delft University of Technology, 2629 HZ Delft, The Netherlands

[3] Tampere University, High Voltage Engineering, P.O. Box 1001, FI-33014 Tampere, Finland

[4] VTT Technical Research Centre of Finland Ltd, P.O. Box 1001, FI-33014 Tampere, Finland

[5] Department of Electrical, Electronic and Information Engineering ''Guglielmo Marconi,'' University of Bologna, 40136 Bologna, Italy

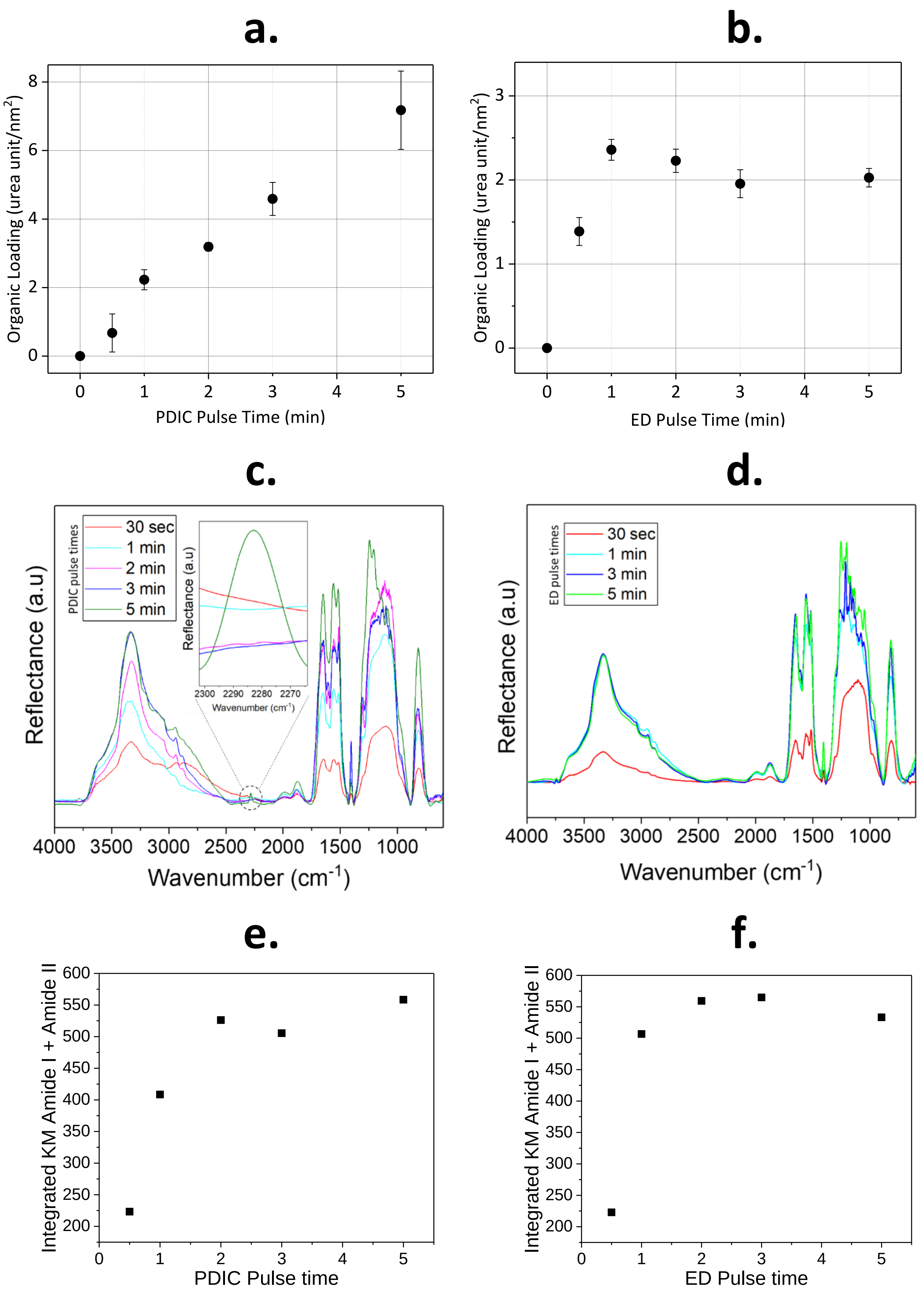
a.
Organic Loading (urea unit/nm2)
PDIC Pulse Time (min)
b.
Organic Loading (urea unit/nm2)
ED Pulse Time (min)
c.
PDIC pulse times
30 sec
1 min
2 min
3 min
5 min
Reflectance (a.u)
Wavenumber (cm-1)
d.
ED pulse times
30 sec
1 min
3 min
5 min
Reflectance (a.u)
Wavenumber (cm-1)
e.
Integrated KM Amide I + Amide II
PDIC Pulse time
f.
Integrated KM Amide I + Amide II
ED Pulse time

Figure S1. Saturating behavior of the polyurea film growth after 5 cycles. This was done by varying the PDIC precursor exposure time, at a constant ED pulse time of 2 min, and vice versa with a constant PDIC exposure time of 1 min. In both cases, N_2 purging times of 10 min were used following each precursor exposure. Saturation curves from TGA: polyurea film growth at different a) PDIC and b) ED pulse times. c) DRIFTS spectra for the saturation experiments with different precursor pulse times. d) Saturation curves from DRIFTS: integrated Kubelka-Munk (KM) amide I and amide II bands.

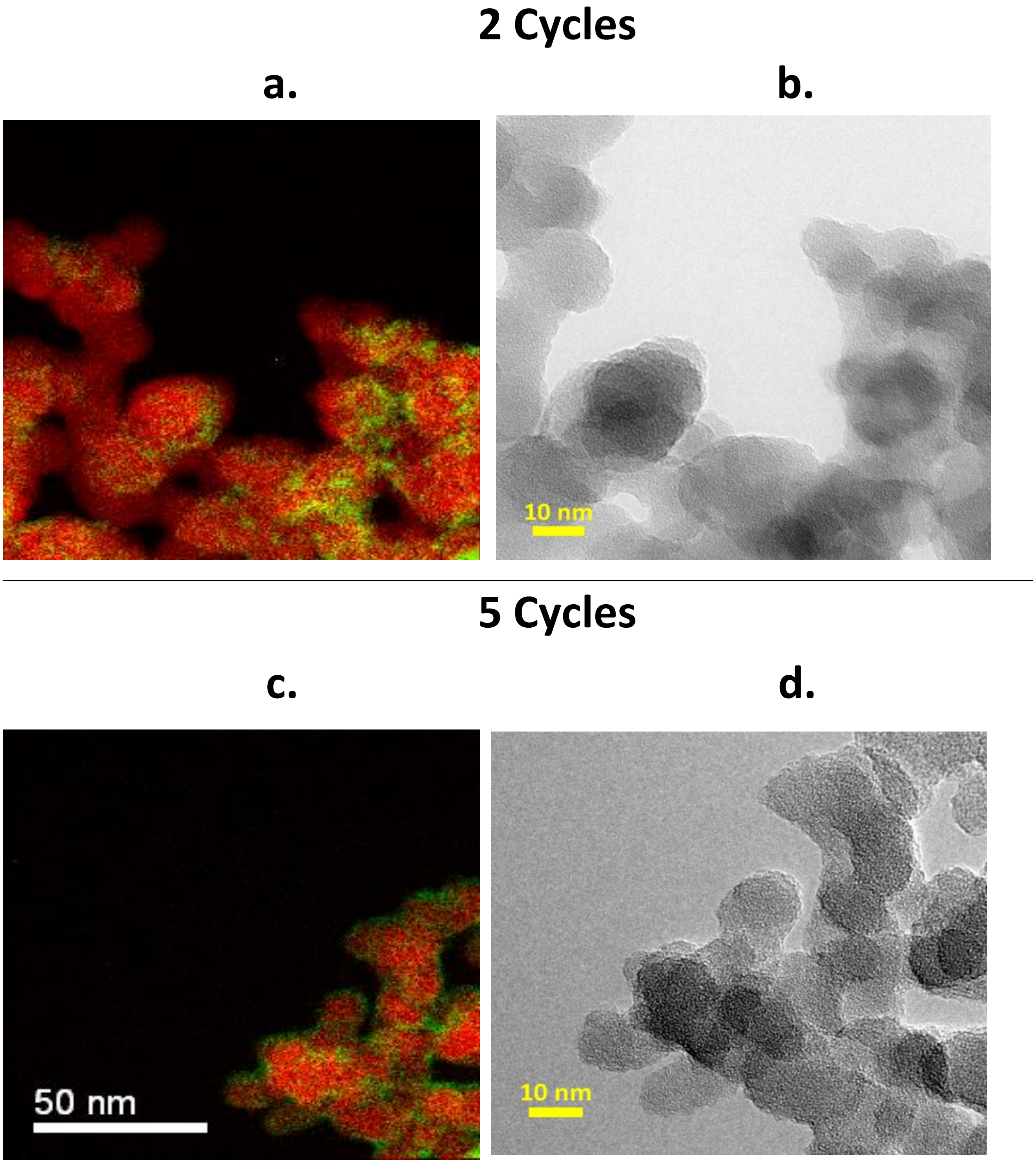

10 Cycles

e. **f.**

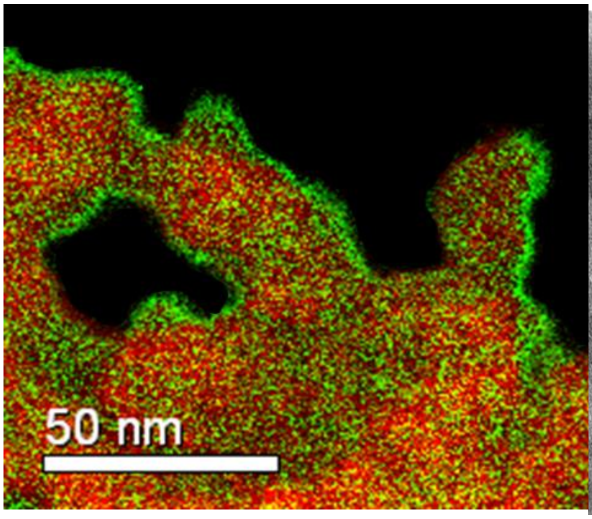

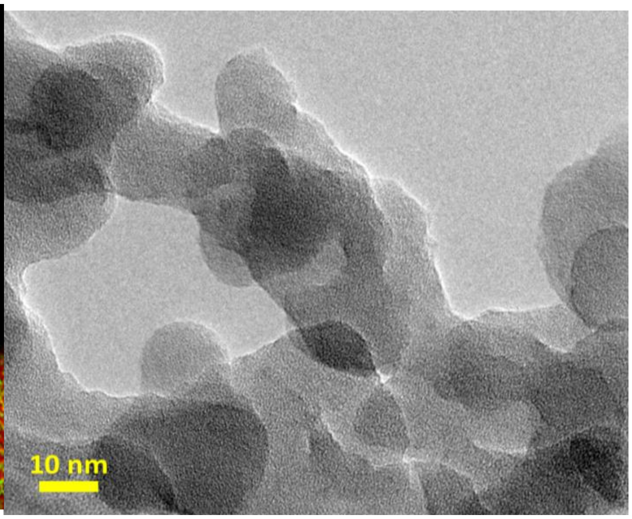

25 Cycles

g. **h.**

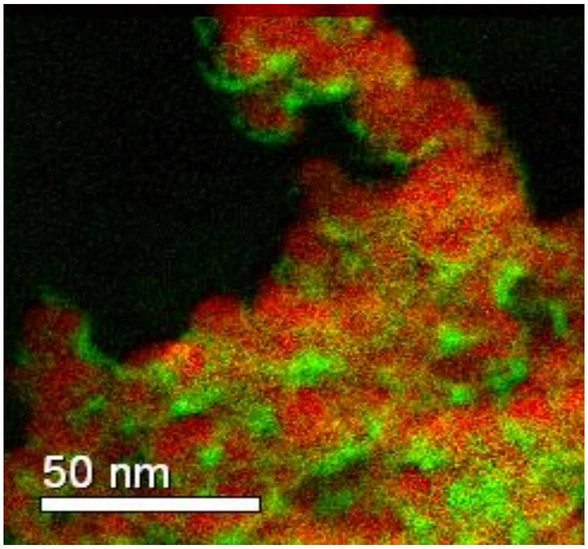

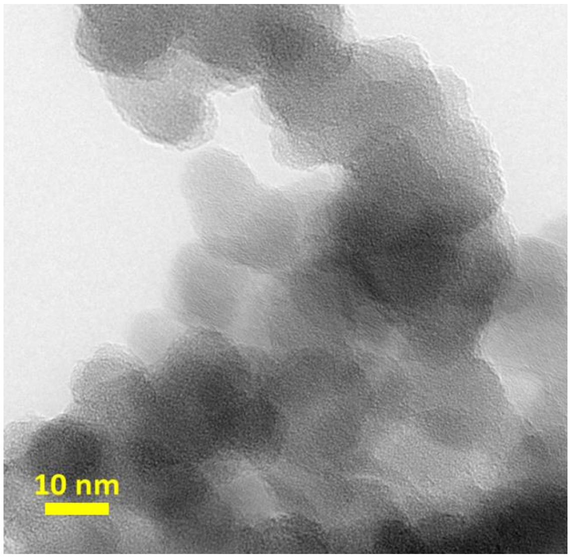

Figure S2. a, c, e, g) Carbon and silicon mapping from TEM images (green: C, red: Si); b, d, f and h) TEM images of the modified NPs. The images demonstrate the spatial distribution of the deposited film on the silica structure.
